# Libraries as Telehealth Hubs: Bridging the Digital Divide and Expanding Health care Access

**DOI:** 10.1089/tmr.2024.0075

**Published:** 2025-01-08

**Authors:** Elizabeth A. Krupinski, Dana Abbey, Mala Muralidaran, Faith Steele, George Strawley

**Affiliations:** 1 Department of Radiology & Imaging Sciences, Emory University, Atlanta, GA, USA.; ^2^ Strauss Health Sciences Library, Network of Library Sciences, University of Colorado Anschutz Medical Campus, Aurora, Colorado, USA.; ^3^ State Library of Arizona in the E-rate and Broadband Branch, National Library of Medicine, Bethesda, Maryland, USA.; ^4^ Network of the National Library of Medicine, University of Maryland Baltimore, Baltimore, Maryland, USA.

**Keywords:** telemedicine, libraries, virtual care symposium

## Abstract

Libraries can play a valuable role in the delivery and support of telehealth services. This article is based on a panel of experts convened for the Virtual Care Symposium 2024, “From Novelty to Sustainability: How to Embed Virtual Care Into the Post-Pandemic Healthcare Delivery Template,” to discuss ways to leverage community spaces to expand telehealth access. Strategies for successful implementation and operation are provided along with insights into possible challenges based on real-world examples. Libraries can play a crucial role in bridging the digital divide by providing internet access and digital devices to the public, particularly in rural and underserved communities.

## Introduction

This report is based on a panel “Leveraging Community Spaces to Expand Virtual care access,” presented at the March 13, 2024, the “From Novelty to Sustainability: How to Embed Virtual Care Into the Post-Pandemic Healthcare Delivery Template” Virtual Care Symposium, with the authors as speakers. Each one presented a perspective based on their involvement with establishing or helping others establish telehealth services in public libraries. The report is not meant to provide a complete overview of the current status of telehealth services in libraries, as there are many other papers that cover the topic in greater detail, but rather to provide readers with a taste of some of the key aspects of the role of libraries in telehealth and ideas about how they can proceed with such a program and where to find some relevant information.

Libraries are becoming increasingly important as telehealth hubs, especially for underserved and rural communities. They offer unique advantages for delivering and supporting telehealth services. The advantages of libraries for telehealth are multifaceted. They provide accessibility and reach as they are centrally located and easily accessible to the public, especially in underserved and rural areas. They provide access to technology and the internet for individuals who lack these resources at home, thereby bridging the digital divide. The wide distribution of libraries across urban and rural locations makes them more accessible than hospitals. Libraries can mitigate known barriers to healthcare, including geographical distances since they are often in small towns and centrally located, transportation challenges since there is reduced need to travel to larger cities to access care, and childcare needs, as many libraries host childcare activities or have play/reading areas for kids.

Libraries offer private spaces for telehealth appointments, ensuring confidentiality and a quiet environment for sensitive conversations. They can offer technology and support, as libraries are equipped with computers, high-speed internet, and other necessary technologies for telehealth. Staff can also provide technical assistance to users unfamiliar with telehealth platforms. Health information resources are readily available, as libraries offer a wealth of books, databases, and access to credible online sources. Librarians can guide users to reliable health information and resources that complement their telehealth consultations.

Essential to health care in general and telemedicine in particular is community engagement and trust. Libraries are trusted community hubs with established relationships with local residents. They can serve as a trusted intermediary between health care providers and the community, promoting telehealth services and encouraging their use. Libraries can host health education programs and workshops to inform the community about telehealth services. They can partner with health care providers to offer joint programs and outreach efforts.

Settings vary by library depending on existing infrastructure or resources to renovate. Some libraries use kiosks, often large enough to accommodate multiple people and those with accessibility needs. Some employ social workers as digital health navigators, providing assistance with a variety of social services and health challenges. Some create private rooms converted from a low-use space. Many have invested in high-quality equipment to enhance the quality of telehealth appointments, often through collaboration with hospital systems, public health offices, academic institutions, or other stakeholders. An ongoing challenge, however, for rural hospitals is access to broadband services, with less than half of rural libraries connected and those that are often having slower speeds than urban counterparts. Libraries have partnered with various stakeholders to offer health fairs, telehealth demonstrations, health screenings, and equipment showcases. Some train health care navigators and recruit retired health care providers to volunteer at libraries. The exact number of libraries that offer telehealth services is not known, but it does appear to be growing considerably.

There are some common key considerations for implementing telehealth services in libraries.

Infrastructure considerations include repurposing existing spaces or developing new spaces for telehealth appointments, ensuring adequate internet speed and reliability, providing necessary equipment such as laptops, cameras, microphones, and basic medical devices, and establishing sanitation protocols; updating existing staffing or finding funding to hire social workers or dedicated telehealth navigators to support the program; and building partnerships with community organizations can be valuable in building a strong team. Flexible scheduling systems are crucial, considering the library’s operating hours and the potential for appointments outside those hours. Clear communication of available appointment times to the community is also important. Ensuring patient privacy is paramount. Libraries should consider using scheduling systems that minimize the disclosure of appointment details and avoid using telehealth computers for non-health-related purposes.

Ensuring physical accessibility for people with disabilities is essential. Libraries should consider the needs of individuals with mobility impairments, visual or hearing impairments, and cognitive disabilities when designing and implementing telehealth spaces and services. Libraries should provide training to staff members on telehealth technology, privacy protocols, and basic troubleshooting. Training for community members on accessing and utilizing telehealth services may also be necessary. Libraries should also explore sustainable funding models, such as partnerships with health care providers, grants, and reimbursements for telehealth services.

According to the American Hospital Association,^1^ there are 6,120 hospitals in the United States, the majority of which are community hospitals in urban areas. By comparison, there are over 17,000 public libraries in the United States (this does not include academic, special, government, or Armed Forces libraries),^2^ with a much wider distribution across urban and rural locations than hospitals. Given the challenges and inequities in access to healthcare that unfortunately exist,^3,4^ organizations are developing creative ways to help patients access providers without having to travel extensively or invest in expensive telecommunications technologies.

Leveraging community spaces such as libraries to expand virtual care access for populations that do not have access to the same resources is critically needed today. Hospitals are generally located in higher population density areas in most states, in and around big cities, with fewer in rural and less populated areas. But if you look at where libraries are located, there are often quite a few more of them in every state, and they are more widely distributed, especially in the rural areas. This presents unique opportunities to reach out to patients and give them the resources, the opportunities, and the locations to access telemedicine resources. The panel discussed several options and programs.

## Perspectives from the Network of the National Library of Medicine Region 4

The Network of the National Library of Medicine Region 4 coordinates health information outreach in Arizona, Colorado, Idaho, Montana, New Mexico, North Dakota, South Dakota, Utah, and Wyoming. They work with community-based organizations, libraries of all types and sizes, and public health departments. Initiatives change based on what current needs are, and current efforts center on health information, bridging the digital divide, which telehealth falls into, and looking at the environmental determinants of health. Even if all households in the United States had high-speed internet, some may still lack a reliable or updated device to connect; they may not have a private space at home to conduct a medical consultation; it may not be a safe place for them to do so, or they might need help navigating technology and finding health information. Enter the library.

Rural libraries often serve populations of less than 25,000 people and are in communities far from medical care. These communities generally do not have robust mass transit, Uber, or Lyft. Offering telehealth services in communities at the library is a great service as an access point. Library staff can help community members efficiently and effectively learn how to look for health information and locate reliable health resources.

When it comes to offering telehealth in libraries, there is no one size fits all. It can be offered in a variety of ways, and many libraries take several different approaches to make sure they are reaching all the needs of the community. At the most minimal level, investment in resources for device lending is required, so many libraries offer hotspots and the ability to check out iPads or laptops so patients can take technology anywhere that is suitable for them to have an appointment.

There may be infrastructure updates required, such as repurposing existing library spaces for telehealth appointments or developing new spaces. Options include a room or a study space converted into a standalone telehealth room, a place to put a kiosk, or multipurpose room where a mobile cart with technology can be rolled in. Key to success is thinking about communities and partners. Depending on the scope of the services, some libraries may need to update existing staffing to support telehealth programs, such as hiring social workers or dedicated telehealth navigators. This is where community partners can be valuable.

There are some good examples of libraries already doing telemedicine. Delaware Libraries have telehealth in many libraries. They work with multiple community members who regard the program as a key asset. The Dover Public Library just put a moveable kiosk up, and it works quite well.

The Kearns branch of the Salt Lake County Library System in Utah has a partnership with the University of Utah Health System that was started as a pilot in 2023. They have high-speed internet and basic examination tools in their “virtual visit” room (e.g., pulse oximeter, scale, blood pressure cuff), so the nurse or physician at the other end of the telehealth call can get basic biomedical data. They also provide library staff to help when available.

In Texas, there is another great service in Pottsboro, where they converted a low-use space for private room visits with customized lighting and other features that can be useful for things such as dermatology appointments, where lighting is critical for seeing skin conditions and rendering diagnoses. The library did have to invest in some high-quality equipment, but it makes the telehealth appointment for the client and provider much richer and provides much better information.

They also partnered closely with the University of North Texas Health Science Center at Fort Worth on the design and delivery. They helped the library understand about cleaning and sanitation of the room and the devices. There was also consideration of best practices for scheduling because they wanted to make sure they were providing a uniform, seamless experience for community members wanting to make an appointment.

## Network of the National Library of Medicine

We found that libraries and librarians wanted training in telehealth specific to libraries and the unique concerns that libraries may have. Thus, we created an asynchronous course called Telehealth 101 on our online platform (https://www.nnlm.gov/training/class-catalog/telehealth-101-what-libraries-need-know). Unlike most telehealth courses, it is not geared towards providers but to libraries and library issues. Sessions are spread over 3 weeks and should take about 3 hours to complete. It also offers credit to the Consumer Health Information Specialization through the Medical Library Association.

The course objectives are broad, covering topics such as space needs, funding, privacy, HIPAA, and liability, as these were the areas that libraries had the most questions around when they were looking at how to start or support their existing telehealth service. It is important to emphasize there is no right way. Each library system, even a branch, can adopt what feels the best for them, and people can be creative with the particular approaches they adopt.

For example, some libraries cannot obtain funding for a telehealth kiosk, but they might have funds for a used mobile cart. Sometimes they build partnerships with other existing health organizations that can provide some infrastructure that the library may not have funding for.

Once we developed the course, we opened registration and sent out an email. It was full in a few days. We ended up offering two sessions of our pilot class as we wanted to limit registration to about 75 people per session to keep it manageable for the instructors.

We had 41 states with a total of 53 people who completed the course, which included the coursework and the final activity. In the final activity, we asked participants to brainstorm ideas specific to their location and specifically how they would approach telehealth for their area. Feedback was positive, with one saying they were so fired up they did not even want to do the evaluation, as they would rather write grants to figure out how to implement telehealth. Attendees also found it useful to see other examples and connect with other library staff that were actively doing telehealth.

There was information about funding possibilities, infrastructure, and even video modules of the existing systems in Delaware, Hawaii, and Pottsboro talking about their experiences setting up their system. Attendees appreciated the real-world experiences of libraries, and it gave them more ideas on how to explore where they would start in their library. The program has continued given the success of the pilots.

There is even a LinkedIn group called the National Working Group for Telemedicine and Libraries. The group is made up of healthcare providers, librarians, public health advocates, and others who aim to improve access to care.

## The Arizona Experience

Libraries in telehealth are essential due to community broadband needs and digital inclusion priorities. There is a significant lack of broadband into homes in most rural areas in Arizona. Childcare, taking time off from work, and other needs can increase the digital gap in areas where getting to a hospital or clinic might take an entire day. Not knowing how to get online to meet with healthcare service providers is a huge challenge.

Libraries can provide equipment and training by library staff and other specialists like student nurses. More importantly, libraries know how to do customer care—librarians are trained customer care specialists. Many libraries have rooms that can be used or repurposed for telemedicine. One of our libraries used their kitchen and converted it into a part-time room for patients to converse with their health care specialists.

At many of our libraries, nurses and nursing staff come on a regular basis to the library to help patrons with behavioral issues, hygiene, immunization, medications, and even getting a valid ID for homeless patrons. As libraries have healthcare databases, these professionals can also access relevant resources to help improve their knowledge and skills.

Many libraries loaned hotspots and iPads to patrons and provided other resources and activities, such as vaccinations and fitness, nutrition, and wellness classes for adults and children. During the pandemic, libraries helped sign people up for the affordable connectivity program (ACP). We have over 22 indigenous tribes in Arizona, and we got many of them signed up for ACP through the library. During the pandemic, we did things such as providing Wi-Fi in parking lots and using our mobile book vans to provide hotspots.

These activities led to us starting two pilot programs within libraries, one in Aho and the other at our Walker libraries, which are very close to the southern border and very far away from healthcare facilities. We developed partnerships with several organizations throughout the state to accomplish all this, including broadband stakeholders. We have a website called Connect Arizona, where we post all the free places where you can get free Wi-Fi. We have partnerships with fire departments and Emergency Medical Technician (EMTs). We have telehealth equipment, which is very novel for our library patrons. We host health fairs, for example, at the Tuba City library to start the service for Native Americans. We host Health Thursdays and Telehealth Tuesdays events with programming for patients to learn about healthcare options and health-related programs on topics such as nutrition, exercise, yoga, speech therapy services, and autism programs. We have telehealth equipment available so patrons can learn to use it and connect with their service providers. We guide people to resources and help them sign up for insurance.

We are collaborating with the American Heart Association to supply the Pima County Library with blood pressure cuffs that they can check out to all branches. We had partners that received an NIH grant to acquire liver scans, but they did not have a place they could do it in, so they used one of our chapter houses on Navajo Nation. The Navajo Nation has high rates of liver cancer, so it was a great site for such services. The libraries helped set up a tent, did all the preliminary data collection and sign-up. Vitals were acquired, and the liver scans were done, and images were interpreted remotely. We expected to do about 25 liver scans, but 85 people signed up, and we had to extend the event to multiple days. This was possible only because we had broadband in that area that was lacking before the partnerships we developed.

## Summary

Libraries are emerging as vital telehealth hubs, particularly for underserved and rural communities, offering accessibility, technology, and privacy for health consultations.^4–10^ With over 17,000 public libraries across the U.S., they are often more readily available than hospitals, helping to bridge the digital divide by providing internet access and technical support. Libraries can serve as trusted intermediaries between healthcare providers and the community, hosting health education programs and partnering with local organizations to enhance telehealth services. Successful implementation requires thoughtful infrastructure planning, staff training, and community engagement, showcasing the adaptability of libraries to meet diverse health needs.

## Abbreviations Used

ACP: affordable connectivity program
EMTs: Emergency Medical Technician
